# System for Tool-Wear Condition Monitoring in CNC Machines under Variations of Cutting Parameter Based on Fusion Stray Flux-Current Processing

**DOI:** 10.3390/s21248431

**Published:** 2021-12-17

**Authors:** Arturo Yosimar Jaen-Cuellar, Roque Alfredo Osornio-Ríos, Miguel Trejo-Hernández, Israel Zamudio-Ramírez, Geovanni Díaz-Saldaña, José Pablo Pacheco-Guerrero, Jose Alfonso Antonino-Daviu

**Affiliations:** 1 CA Mecatrónica, Facultad de Ingeniería, Campus San Juan del Río, Universidad Autónoma de Querétaro, Av. Río Moctezuma 249, San Juan del Río 76807, Mexico; ayjaen@hspdigital.org (A.Y.J.-C.); raosornio@hspdigital.org (R.A.O.-R.); mtrejo@hspdigital.org (M.T.-H.); iszara@doctor.upv.es (I.Z.-R.); gdiaz17@alumnos.uaq.mx (G.D.-S.); jpacheco10@alumnos.uaq.mx (J.P.P.-G.); 2 Instituto Tecnológico de la Energía, Universitat Politècnica de València (UPV), Camino de Vera s/n, 46022 Valencia, Spain

**Keywords:** condition monitoring, tool wear, cutting speed, feed rate, sensors fusion, stray flux, ac current

## Abstract

The computer numerical control (CNC) machine has recently taken a fundamental role in the manufacturing industry, which is essential for the economic development of many countries. Current high quality production standards, along with the requirement for maximum economic benefits, demand the use of tool condition monitoring (TCM) systems able to monitor and diagnose cutting tool wear. Current TCM methodologies mainly rely on vibration signals, cutting force signals, and acoustic emission (AE) signals, which have the common drawback of requiring the installation of sensors near the working area, a factor that limits their application in practical terms. Moreover, as machining processes require the optimal tuning of cutting parameters, novel methodologies must be able to perform the diagnosis under a variety of cutting parameters. This paper proposes a novel non-invasive method capable of automatically diagnosing cutting tool wear in CNC machines under the variation of cutting speed and feed rate cutting parameters. The proposal relies on the sensor information fusion of spindle-motor stray flux and current signals by means of statistical and non-statistical time-domain parameters, which are then reduced by means of a linear discriminant analysis (LDA); a feed-forward neural network is then used to automatically classify the level of wear on the cutting tool. The proposal is validated with a Fanuc Oi mate Computer Numeric Control (CNC) turning machine for three different cutting tool wear levels and different cutting speed and feed rate values.

## 1. Introduction

The manufacturing industry has been a fundamental and highly relevant sector in the economic development of many countries [1]. In this regard, the computer numerical control (CNC) machine has taken a fundamental role and is involved in many manufacturing industries. In order to obtain an increased quality production, along with maximum economic benefits (one of the main objectives pursued by the involved companies), industrial processes must be optimized by means of the different factors that can strongly influence manufacturing expenses, such as cutting tool costs, electricity costs, machining efficiency, and machined surface quality [2]. As reported in [3,4], the costs of machining and replacing the cutting tool can represent up to 25% of the total production costs, and even a slight reduction of machine cutting tool downtime improves the production rate significantly [5]. These figures and facts highlight the necessity of using cutting tools in excellent condition without replacing them unnecessarily. However, cutting tools are subjected to constant stresses, which leads to an imminent and gradual wear that tends to deteriorate the machining quality and cutting efficiency, no matter the tuning of optimal cutting parameters. Additionally, this situation may lead to the shutdown of the machine tool in severe cases, with approximately 20% of the production time being wasted in this kind of downtimes [6,7]. In this regard, it is essential to develop tool condition monitoring (TCM) systems and methodologies capable of extracting relevant information from the machining process and its different elements and signals in order to effectively correlate and diagnose the healthiness state of the cutting tool and achieve adequate maintenance actions. Currently, most of the TCM methodologies have relied on information obtained from different physical magnitudes. Each technique is subjected to its own constraints and requirements, especially those related to intrinsic implications due to the sensors used: reduced space for installation, invasiveness to the machining process, and high sensitivity to environmental noise, among other factors. As a consequence, the feasibility of applying diverse methods in real operating conditions is directly affected and limited according to the sensors used and their characteristics [8].

With regards to CNC cutting tool wear research, it should be noted that there are two main trends: the first oriented to optimizing the machining cutting parameters in order to take care of the tool wear, and the second relying on methods focused on detecting the tool wear. For example, the work carried out in [9] demonstrates that the cutting speed has the most significant effect on tool wear, followed by the variation in the feed rate and finally the depth of cut. Similarly, Kuntoğlu et al. [10] presented a method that deals with the variation of three parameters: cutting speed, feed rate, and tool tip, in order to determine the most optimal parameters that reduce progressive tool wear.

On the other hand, TCM methods have been developed over the last few decades that can be divided into two main groups: (a) direct techniques and (b) indirect techniques. The most preferred are the indirect techniques because they do not require the machine to be stopped, and the final diagnosis is performed by signal collection, signal processing, and classification, which relates then to the observed values of cutting tool wear [11]. In this context, several investigations have been carried out that have been able to properly correlate diverse physical magnitudes to the cutting tool wear at a certain level of uncertainty. Therefore, vibration signals, cutting force signals, acoustic emission (AE) signals, temperature signals, motor current signals, and image processing, among other factors, have been extensively used for these purposes in several investigations [8,12,13]. Vibration is considered one of the variables that best reflects the TCM process. Certain factors, such as the fact that it is not necessity to make any changes to the machine tool or workpiece fixtures, are among the most important advantages that distinguish vibration measurement [14]. However, as vibrations are generated during machine operation, even when the tool is not engaged in cutting, effectively distinguishing between entity-cut and air-cut operations is still an open challenge [13], and it is a constraint that limits its applicability. In addition, the position at which the sensor is installed and the use of cutting fluid can affect the vibration signals [15], making them difficult to be filtered; therefore, vibration signals are likely to provide inaccurate data [16]. On the other hand, force sensors and signals have been demonstrated as being very sensitive to changes in the tool condition and can therefore accurately estimate the tool state [17]. However, cutting force sensors are difficult to apply in industrial environments because their physical properties limit the physical size of a workpiece, which is not appropriate when milling medium and large workpieces [18]. Moreover, the problem that cutting force monitoring interferes with the motion control of the spindle and stage in a milling machine has been pointed out [19], along with the fact that commercial dynamometers are very expensive. In turn, AE signals are known to have a superior sensitivity compared to cutting force and vibration signals. They are also non-invasive and propagate at a frequency much higher than the characteristic frequency caused by cutting, which reduces interference [20,21]. Nevertheless, intermittent cutting during the milling processes results in AE signal spikes, which complicate the signal analysis and processing stage [18]. Additionally, AE sensors are highly sensitive to environmental noise, which complicates the task of extracting valid signal feature information [22]. Similarly, in infrared image-based techniques, any thermal interference can deteriorate the final diagnosis, so the coolant liquid used and hot chips can lead to false indications [23], limiting the application of such methods to specific operating conditions. On the other hand, current signals have been demonstrated as being feasible for diagnosing cutting tool wear due to the fact that cutting force increases with increasing tool wear, and hence the current drawn by the machine motor undergoes a corresponding increase [24]. However, motor currents have the disadvantage of containing a certain amount of noise [19]. In this regard, a very recent investigation, which is the preliminary work of this paper, proposed the analysis of spindle-motor stray flux signals [25]. In that work, the feasibility of such signals was demonstrated; it is a non-invasive technique that does not require the installation of sensors near the working area, and it properly correlates the cutting tool wear under different depths of cut. However, it does not take into consideration the variation of other cutting parameters, which is a very important aspect that must be taken into account because the variation of such parameters is central in real industry applications. Generally speaking, the essential use of sensors installed near the working area that indirectly affect the final results is one of the main limitations in indirect methodologies. As discussed in [13], the use of multiple sensors can achieve better results over a wide range of cutting parameters and cutting conditions than those obtained with a single sensor. In this regard, Huang et al. [26] proposed the combined analysis of force, torque, and vibration signals. They obtained an accuracy above 88.89% under different cutting parameters: feed rate, axial depth of cut, and radial depth of cut. However, as pointed out in [13], there is an increased interference in the milling process associated with multiple sensors (mainly invasive sensors such as torque, vibration, and force sensors), which certainly limits the viability of some sensor fusion techniques. Nevertheless, this problem may be overcome if the fusion information of non-invasive sensors is used. For example, Zhou et al. [27] demonstrated the performance achieved when combining the three spindle-motor current signals (by means of three different time, frequency, and time-frequency domain parameters) for tool-wear condition monitoring under the variation of spindle speed, depth of cut, and feed rate. The authors demonstrated that results with very small errors could be accomplished. Additionally, Hassan et al. [28] proposed an innovative methodology for the diagnosis of cutting tool wear in milling machines by using spindle-motor current, voltage, and power signals, and the empirical mode decomposition (EMD) method. They achieved an accuracy of 98% for detecting the tool condition under different cutting parameters (feed rate, axial and radial depth of cut, and tool diameters) and flank wear levels. Accordingly, as has been shown in various investigations reported in the literature, and as discussed above, the combination of different signal information is an excellent alternative for diagnosing the cutting tool wear in a machining process more reliably and under diverse variations in cutting parameters. Therefore, it would be of great relevance to combine the information provided by different signals (preferably those that are non-invasive to the working area and the machining process) by means of a suitable technique able to extract the most relevant data of each one. Unfortunately, there is no research in the literature focused on the fusion of spindle-motor stray flux and current signals for the diagnosis of cutting tool wear under different cutting parameters, despite such signals being proven to yield comparable results to those of well-established methods with high reliability and the advantage of it being non-invasive, low cost, and immune to some electric motor fault diagnosis [29].

The main contribution of this paper is the development of a non-invasive system capable of monitoring and diagnosing cutting tool wear during a machining process under the variation of two cutting parameters: feed rate and cutting speed. The proposed method relies on the merging of the information provided by stray flux signals (captured around the spindle motor) and spindle-motor currents, which is non-invasive because the sensors are installed away from the machining process and cutting tool area. Additionally, in order to have a sensor that fulfills the requirement of capturing the different stray flux magnetic components at a single point, no matter the position, it is proposed that a triaxial stray flux sensor (made up of three primary hall-effect sensors placed on relatively perpendicular axes) be installed on the frame of the spindle motor. The proposal relies on the computation of selected statistical and non-statistical time-domain features, which are capable of providing information regarding general trends and characteristics concerning the dynamic behavior of a signal. Subsequently, these features are reduced to two main components by means of a linear discriminant analysis (LDA) in order to combine the most relevant information regarding all the captured signals and features. An artificial neural network is then trained and incorporated in order to obtain an automatic classification. The experiments are carried out using a Fanuc Oi mate CNC turning machine considering five different cutting tool wear states: from new cutting tool to severe cutting tool wear. The results achieved show that the proposed method is able to diagnose and correlate the cutting tool wear to the captured spindle-motor stray flux signals, regardless of variation in the cutting parameters used during the machining process. The proposal may find great applicability in several machining processes that require constant cutting tool monitoring.

## 2. Materials and Methods

### 2.1. Stray Flux Signal Analysis

Magnetic stray flux analysis is an emerging technology that has gained great relevance in the field of electrical machine fault diagnosis in recent years, especially for the analysis of induction motors. This is mainly due to its excellent characteristics and performance. Additionally, it is a non-invasive technique that requires low-cost sensors, and it has been shown to be effective in situations where conventional methods generate false diagnoses [29]. The techniques based on the analysis of stray flux rely on magnetic flux that radiates outside the frame of the machine and is induced by stator and rotor currents; this flux is modified when a fault is present and is dependent on the fault origin: electrical, mechanical, and even those faults related to the kinematic chain to which the motor is connected, as demonstrated in several investigations [30]. In this way, when an electric motor is operating under a fault condition, this can be reflected in the rotor/stator current signals, which in turn further modify the magnetic stray flux captured around the motor according to the fault origin.

**Figure 1:**
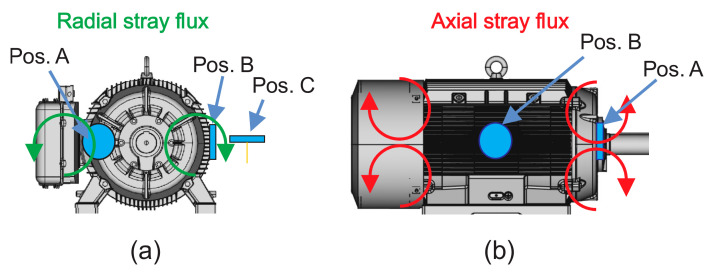
Magnetic stray flux components: (**a**) radial stray flux; (**b**) axial stray flux.

**Figure 2:**
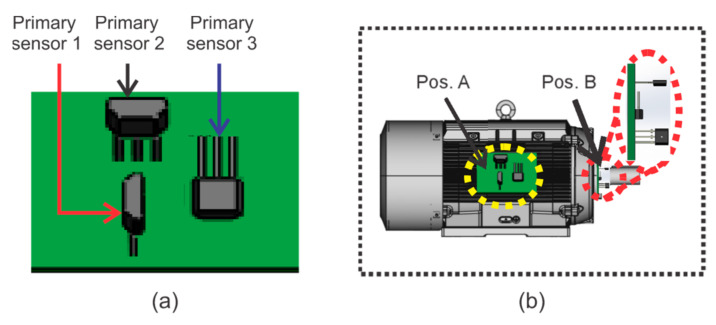
Triaxial stray flux architecture: (**a**) primary sensors; (**b**) axial stray flux installation.

The stray flux can be analyzed through its two magnetic components: axial and radial [31]. It is known that the axial magnetic field is generated by currents in the stator end-windings or rotor cage end ring and is located in a plane that comprises the machine axis, while the radial magnetic field is located in a plane perpendicular to the machine axis. Furthermore, as reported in [30], based on the presumed circulation of the field lines of the axial and radial stray fluxes, it is possible to capture such stray flux components separately by installing suitable sensors in specific positions around the motor frame. Then, as shown in Figure 1, if a proper magnetic coil-based sensor is installed as in position A, the axial field can be measured; the radial field can be acquired if a sensor is installed as in position C, as shown in Figure 1, and finally by installing a sensor as in position B, the combination axial + radial stray flux can be captured. Alternatively, it is possible to use a triaxial magnetic flux sensor with three individual sensitive devices (primary sensor 1, primary sensor 2, and primary sensor 3) placed on perpendicular axis in order to capture the three orthogonal components of the magnetic flux, as shown in Figure 2a. In this way, this triaxial sensor can be placed at different points around the frame of the machine to capture the different stray flux components, as shown in Figure 2b.

### 2.2. AC Current Demand of the Spindle Motor in the CNC Machine

One of the most important electromechanical elements of a CNC machine is the motor installed in the spindle. Thus, it is typical that a three-phase induction motor manages the spindle by means of a variable frequency drive in order to reach the rotation speed defined by the CNC that is required for the machining of a workpiece. It is well known that the AC current demand by the spindle motor is a good indicator of the anomalies presented during the machining process. Therefore, the measurement of the AC current demand from the spindle motor is crucial in applications where tool-wear conditions need to be detected. Normally, the AC current demand by the spindle motor is detected and acquired through commercial current clamps because they are non-invasive sensors, and there exist models that use a flexible Rogowski coil for allowing the sensors to make measurements in a very simple way, as in the case of this work.

### 2.3. Cutting Parameters in CNC Machines

The cutting parameters are commonly selected depending on the materials of the workpiece (aluminum, iron, and others) and available cutting tools (cermets, nitride, carbides, covered carbides, and others). A deficient cutting parameter selection can provoke tool wear, tool failures, vibration, high temperatures, and incorrect surface rugosity, among other things. For the selection of cutting parameters in the turning process, the feed rate (*f*) and the revolution per minute of the spindle (*N*), as programming parameters in G-code, should be accounted for. Both parameters can be related according to Equations (1)–(3). Additionally, if an aluminum alloy is to be machined in the same was as other materials, particularly in the roughing or finishing process, the parameters consideration for avoiding the effects stated previously, can be found using the tables in [32] as a guide.

The cutting speed ($v_{c}$) or feed rate, can be proposed according to those tables. Equation (1) can relate those parameters.

(1) $$v_{c}=\pi D_{o}N$$

where $v_{c}$ is the maximum cutting speed in m/min, $D_{o}$ is the external diameter of the workpiece in mm, and N is the spindle rotation in rpm. Depth of cut ($a_{p}$), in mm, can be obtained from the relation shown in Equation (2).

(2) $$a_{p}=\frac{D_{o}-D_{f}}{2}$$

where $D_{f}$ is the final diameter of the workpiece after a faced machining process. Programming the G-code and depth of cut parameter should depend on the radial or diametral programing mode in the respective CNC machine.

On the other hand, the feed rate in mm/rev can be calculated with Equation (3).

(3) $$f=\frac{v_{f}}{N}$$

where $v_{f}$ (given in mm/min) is the axial speed of the cutting tool.

**Figure 3:**
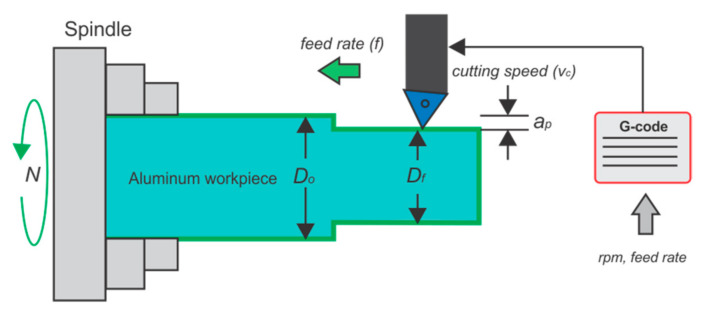
Machining process and cutting parameters.

Equations (1)–(3) can relate easily, if two parameters are holding or settled in certain desired constant values, for computing the other cutting parameters. The parameters mentioned here can be seen in Figure 3.

### 2.4. Tool-Wear Calculation

**Figure 4:**
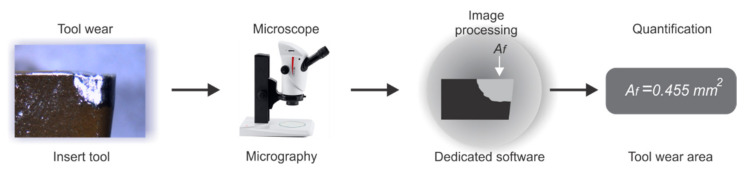
Tool-wear area quantification.

Tool wear has been widely studied in the literature. The wearing process is complex due to the chaotic behavior between workpiece and cutting tool. High stresses, temperatures, sliding of chips, and constant repetitions of the machining process, among other factors, are some the causes of this complexity. Tool-wear classification includes flank wear, crater wear, nose wear, or tool failure. Diverse methods for measurement of the tool wear can be applied, including direct inspection, image processing, and microscopy. Image processing for tool-wear area computing as quantification can also be applied. As described in [12], the measuring of tool wear in cutting tools can be considered as area, length, or width. In this work, with the aim to validate the developed method, the tool-wear area is used and described by $A_{f}\;$in ${\mathrm{mm}}^{2}$, and it can be computed following the procedure shown in Figure 4. A micrography from the insert, with tool wear, is taken and processed using dedicated software for counting the pixels in the zone where tool wear appears. Corresponding conversion to ${\mathrm{mm}}^{2}$ is considered and reported as a tool-wear area.

### 2.5. Statistical Time-Domain Features

As has been shown in diverse investigations in the literature, a system with different operating conditions may have signals with varied statistical parameters. In this way, it is expected that a system working under a fault condition will reflect its status on some statistical parameters [33]. Statistical time-domain indices such as mean, root mean square (RMS), standard deviation, variation, impulse factor, and shape factor, among others, have been successfully used for the condition monitoring of electric machines [34]. In addition, time-domain features have proliferated in the area of electric fault diagnosis, especially in schemes intended for online analysis due to their excellent characteristics and benefits, such as low computation burden and memory resources required to compute them, their capability to provide information regarding general trends, and their simplicity [35]. In this way, a time-domain signal may be successfully characterized by a set of several features. In this work, a set of 15 statistical time-domain features ($T_{1},\;T_{2},\ldots ,T_{15}$) is considered, whose mathematical formulation is described in Table 1, from Equation (4)–(18), where $x_{i}$ is the time-domain signal for sample i=1, 2, …, N, with N being the number of data points.

### 2.6. Fractal Dimension Analysis

Fractal dimension (FD) analysis is a concept of chaos theory that attempts to measure the amount of self-similarity or repeated patterns that can be found in a time-domain signal [36]. The fractal dimensions of a signal may range from 1.0, for straight lines, to 2.0, according to the complexity and self-similarity [37]. In this way, an FD analysis may be useful to assess the regularity of a time-domain signal. This implies that any variation in the waveform regularity may yield information related to the dynamic behavior of the system under analysis. Therefore, extrapolating these ideas in the case of cutting tool wear in CNC machines, it is expected that the spindle-motor stray flux and current signals may suffer an alteration due to cutting tool wear (i.e., transient characteristics in the measured signal may change), which can vary according to the severity of the damage; such change can be characterized by a fractal index. In this work, two fractal indexes are evaluated: Katz’s FD (KFD) and Higuchi’s FD (HFD).

#### 2.6.1. Katz’s Fractal Dimension (KFD) Computation Procedure 

The mathematical procedure to obtain KFD is as follows [38]:

Step 1. Find the maximum Euclidean distance, d, between the first sample, $x_{1}$, and sample $x_{k}$ (for k=1, ⋯, N); N represents the number of samples found in the time-domain signal).

Step 2. Obtain the arithmetic sum of the Euclidean distances (L) between successive samples of time-domain signal x and then calculate its average (a) as follows:

(19) $$L=\overset{N}{\underset{k=2}{\sum }}distance\left(x_{k}-x_{k-1}\right)$$

(20) $$a=\frac{L}{N-1}$$

Step 3. Compute the fractality, *KFD*, of the time-domain series signal according to Equation (21):

(21) $$KFD=\frac{\log\left(L/a\right)}{\log\left(d/a\right)}\;$$

#### 2.6.2. Higuchi’s Fractal Dimension (HFD) Computation Procedure

The following steps describe the mathematical procedure to estimate the HFD fractality of a time-series signal [33,35]:

Step 1: Decompose the original time-series signal, x, with  N  samples in new signals or time-domain sequences, $x_{k}^{m}$.

(22) $$x_{k}^{m}=x_{m},\;x_{m+k},\;x_{m+2k},\ldots ,\;x_{m+⎣\frac{N-m}{k}⎦k}\;\;\left(m=1,2,\ldots ,k\right)\;$$

where k and m are integer numbers that determine the time delay between successive samples and the initial sample time, respectively. The $⎣\frac{N-m}{k}⎦$ term denotes a rounding to the integer part of a value.

Step 2: Compute the normalized average length ($L_{m}$) for each sequence, $x_{k}^{m}$.

(23) $$L_{m}\left(k\right)=\frac{\left(N-1\right)}{k⎣\frac{N-m}{k}⎦}\overset{⎣\frac{N-m}{k}⎦}{\underset{i}{\sum }}\left|x_{m+ik}-x_{m+\left(i-1\right)k}\right|\;$$

where the term $\frac{\left(N-1\right)}{k⎣\frac{N-m}{k}⎦}$ is a normalized factor of each sequence.

Step 3: Compute the total length $L\left(k\right)$ by averaging the length of all sequences, $L_{m}\left(k\right)$, for a given k value as follows:

(24) $$L\left(k\right)=\overset{k}{\underset{m=1}{\sum }}L_{m}\left(k\right)$$

Step 4: Modify k=k+1. If $k<k_{\max\;}$ and repeat steps 1 to 3. The $k_{max}$ value is selected to be the number when the slope of the best line fitted to the diagram of the plotted plane ($ln\left[L\left(k\right)\right]$ versus $ln\left[1/k\right]$) remains constant. The obtained slope of this line represents the Higuchi’s fractality value (HFD) of the analyzed signal; Equation (24).

### 2.7. Dicrete Wavelet Transform Energy ($\gamma_{DWT}$)

**Figure 5:**
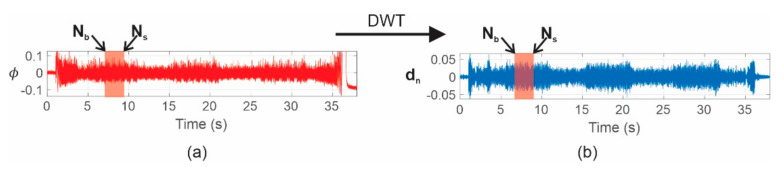
Rectangular window considered to compute $\gamma_{DWT}$ by analyzing the wavelet signal, $d_{n}$: (**a**) stray flux captured by the sensor. (**b**) wavelet signal containing the majority of the fault frequency component.

The discrete wavelet transform (DWT) energy ($\gamma_{DWT}$) is a normalized indicator that provides a general idea of the energy changes in a given signal. This indicator has been widely applied with great success to the diagnosis of faults in electric motors due to its high capacity to evaluate the energy of a specific frequency band. This indicator is suitable for measuring the amplitudes in a band-limited frequency in which a fault component appears, and it is supported by the fact that energies of the faulty band wavelet component increase as the fault severity does; for the case in which the stray flux is analyzed, it relates the stray flux (*ϕ*) captured by the sensor to that of the wavelet signal containing the majority of the fault frequency component ($d_{n}$). This indicator has been suggested in previous works for the detection of some electric motor failures, such as rotor problems [39], and it is adapted here for the purposes of this paper. This normalized indicator is given by Equation (25) (where $N_{b}$ is the position of the first sample to be considered and $N_{s}$ is the last sample covering the time interval under consideration). Additionally, Figure 5 shows an example of a time window considered for the computation of the $\gamma_{DWT}$ index.

(25) $$\gamma_{DWT}=10\;\cdot \log\left[\frac{\sum_{j=N_{b}}^{N_{s}}\phi {\left(j\right)}^{2}}{\sum_{j=N_{b}}^{N_{s}}d_{n}{\left(j\right)}^{2}}\right]$$

### 2.8. Wavelet Entropy ($S_{WT}$)

Due to the uncertainty principle that is inherent in some time-frequency transformations, a critical limitation arises when a specific window is applied to a data series, as in the case of the wavelet transform; if the window is too narrow, the frequency resolution will be poor, and if the window is too wide, the time location will be less accurate. This limitation becomes relevant when the signal has transient components located in time, such as the components in most real signals [40]. To overcome these limitations, a parameter based on the entropy of a signal has been defined from a time-frequency representation provided by the DWT [41]. Hence, the wavelet entropy is intended to determine the amount of order or disorder existing in a signal, that is, it can provide additional information regarding the underlying dynamic processes associated with it. The total wavelet entropy ($S_{WT}$) is given by Equation (26) [42].

(26) $$S_{WT}\equiv S_{\mathrm{WT}}\left(p\right)=-\overset{n}{\underset{j=m}{\sum }}\ln p_{i}$$

where m and n are the first and the last considered wavelet decomposition levels, respectively, and $p_{i}$ represent the relative wavelet energy normalized values, which can be computed by applying (27):

(27) $$p_{i}=\frac{E_{i}}{E_{tot}}$$

where $E_{i}$ and $E_{tot}$ are the energy of wavelet level decomposition, i, and the total energy of all wavelet level decompositions, as given by Equations (28) and (29), respectively.

(28) $$E_{i}=\sum_{k}{\left|C_{i}\left(k\right)\right|}^{2}$$

(29) $$E_{tot}=\underset{i}{\sum }E_{i}$$

## 3. Proposed Methodology

**Figure 6:**
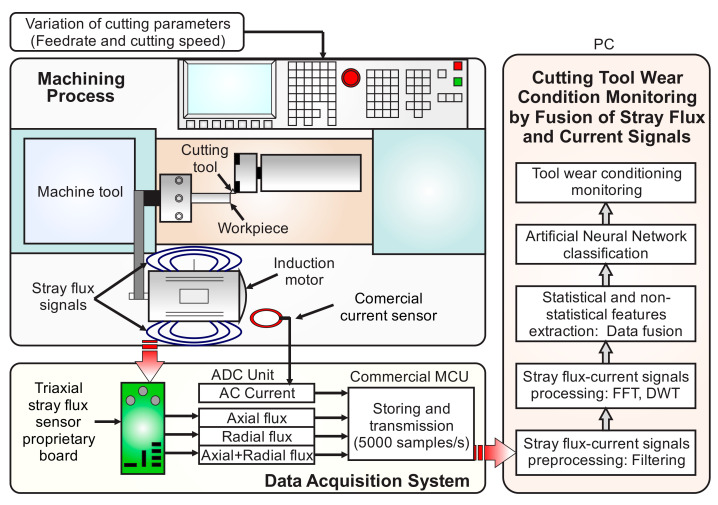
Block diagram of the proposed methodology for tool-wear condition monitoring.

This section presents the procedure to implement the system for tool-wear condition monitoring through signal analysis by fusing the current and stray flux generated over the spindle motor of a CNC lathe machine under variations of the cutting parameters: cutting speed and feed rate. The proposed approach is observed in the block diagram of Figure 6. Note that there are three main blocks for the implementation of the monitoring system, namely: (i) Machining Process, (ii) Data Acquisition System, and (iii) Cutting Tool Wear Condition Monitoring by Fusion of Stray Flux and Current Signals.

In the Machining Process block, the cutting parameters in the CNC lathe for driving the cutting tool to the workpiece under different conditions of cutting speed and feed rate are established: these will be detailed next in the experimental setup. As a result, the spindle motor of the machine generates stray flux and current signals related to the variations of the cutting parameters and the condition of the tool. 

On the one hand, the magnetic stray flux generated around the spindle motor is detected by means of a proprietary board integrating three sensors that conform a non-invasive triaxial sensing module. This board is mounted on the spindle-motor frame of the CNC machine, and it is capable of detecting, simultaneously, the flux coming from the axial, radial, and axial + radial directions, unlike other approaches that use a one directional single sensor. Meanwhile, the signal from the AC current demand by the spindle motor is measured by means of a current sensor integrated by a commercial current clamp. Later, the stray flux and the current signals detected by the sensors are acquired through the ADC of a commercial microcontroller unit (µCU) and transmitted to the PC for processing and analysis. Together, the triaxial stray flux sensor proprietary board, the integrated commercial current clamp, and the commercial microcontroller define the Data Acquisition System (DAS) block.

Once the DAS module has transmitted the acquired signals to the PC, the third main block, known as Cutting Tool Wear Condition Monitoring by Fusion of Stray Flux and Current Signals, is finally run. As a first stage, the sampled data are preprocessed by applying fast Fourier transform (FFT) and a low-pass filter in order to clean the signals of noise effects. The FFT is applied in order to obtain the fundamental frequencies of the acquired signals, because a variable frequency drive (VFD) drives the spindle motor of the machine. Therefore, taking these frequencies into account, the cutoffs for the low-pass filters are defined between 30 Hz up to 200 Hz. Immediately, in a second stage, the filtered signal is then decomposed by the DWT and employed to obtain the statistical and non-statistical indicators. Thereby, in the third stage, the statistical indicators are computed using the expressions of Table 1 through Equations (4)–(18). In counterpart, the non-statistical indicators, such as Katz’s Fractal Dimension (KFD), Higuchi’s Fractal Dimension (HFD), DWT energy ($\gamma_{DWT}$), and Wavelet entropy ($S_{WT}$), are computed through expressions (21) and (24–26), respectively. As a result, a total of 19 indicators are considered in the experiments for generating a general features matrix, as detailed in the experimental setup. The sensor fusion starts from this stage, because the 19 indicators are computed for the signals of the four sensors (AC current, axial flux, radial flux, and axial + radial flux), and all these indicators are structured in a single matrix of features. In the fourth and final stage, this matrix of features is reduced, by applying Linear Discriminant Analysis (LDA), into a 2D representation that separates the clusters of every condition detected. Here, the sensor fusion is completed, because the matrix reduction is made by taking into account the values of all the features in the matrix without discriminating if the features are from the AC current sensor or from the stray flux sensors. Finally, the reduced indicators are employed to perform a classification of the tool-wear condition through an Artificial Neural Network (ANN). For this work, a simple structure of the ANN was selected because its concept and structure are very well-known, it does not require expert knowledge in its use or interpretation, and it can be easily automated and adjusted for the tool-wear classification problem. Thus, due to the facility and low complexity in the classification task for detecting the tool-wear condition after applying the reduction of the matrix of features through LDA, a simple ANN is proposed to keep the computational effort and the number of resources as low as possible. This procedure is executed for both variations of the cutting parameters: cutting speed and feed rate.

## 4. Experimentation

### 4.1. Experimental Setup

**Figure 7:**
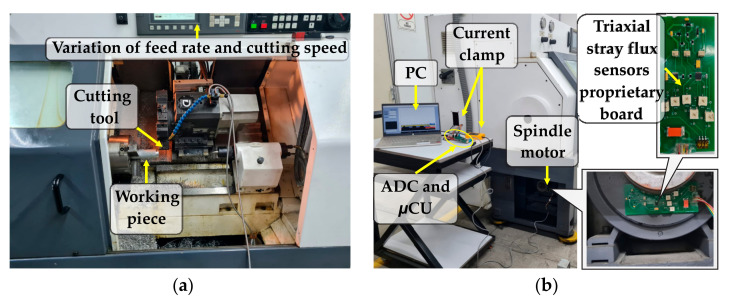
Experimental setup: (**a**) machining process and (**b**) data acquisition system module.

This section describe in detail the experimental setup considered for the tests. In this work, the machining process, observed in Figure 7a, was carried out on a Fanuc Oi Mate CNC lathe (model Realland Smart Motors P96AR06D4152704) that has a three-phase induction motor installed in the spindle with four poles with a rated power of 3.7 kW that is fed at 220 Vac through an VFD. Regarding the data acquisition system module observed in Figure 7b, it is based on the components detailed as follows: A triaxial stray flux sensor proprietary board, which integrates three individual hall-effect sensors, model A1325 from the microsystems brand ALLEGRO^TM^. Each flux sensor mounted on the board has a sensitivity of 5 mV/G, a range of operating temperature between −40 °C and 150 °C, and a bandwidth of 17 kHz (−3 dB), according to the manufacturer data sheet. The overall characteristics of the board are proportionality of the output to the magnetic flux density, low level noise at the output, and immunity to mechanical stress. The flux sensors are placed on perpendicular axes between them in order to capture the magnetic flux from the axial, radial, and combination axial + radial directions, no matter the relative installation of the board in the spindle-motor frame. On the other hand, the AC current demand, also from the spindle motor, is detected by means of a current sensor integrated by a commercial current clamp, model Fluke i3000s, which has three adjustable output ranges of 30 A, 300 A, and 3000 A. Its operating bandwidth is from 10 Hz to 50 Hz, according to the manufacturer data sheet. The current clamp is a flexible Rogowski coil that facilitates the measurements in wires up to 7 inches placed in areas that are difficult to access. Then, the four signals of interest (the AC current and the stray flux from the three directions) are the instantaneous values captured and transmitted to the PC for normalization and processing using the 14-bit ADC of the µCU from Texas Instruments, operating at 5000 samples per second ($f_{s}=5$ kHz) in order to ensure the acquisition of an adequate number of samples.

### 4.2. Study Cases

In the reported literature, the majority of the studies regarding tool-wear condition typically consider the depth of cut for their analysis. For that reason, in the proposed approach, the experimental tests were reinforced by carrying out variations in the cutting speed and also in the feed rate. Those variations are specified in the corresponding ranges that are recommended for machining aluminum 6061 workpieces and that can be found in [29]. Thus, for each trial, the machining entails a cycle of three continuous cuts along the workpiece during 30 s, generating 150,000 samples for the $f_{s}$ specified, observed in Figure 3.

Regarding the cutting tools employed for the tests, a total of six coated carbide inserts, BOEHLERIT TCMT—16T308—MP LCP—25T, were used; these were divided into two groups of three inserts with gradual progressive wear. The first group was used for the analysis in the variation of cutting speed, and the second group was used for the analysis of the variation of feed rate. Then, for each group, three different states were handled: new tool, medium tool wear, and excessive tool wear, calculated as explained in Section 2.3. Therefore, two main sets of experiments were defined.

#### 4.2.1. Study Case: Variation in Cutting Speed

**Figure 8:**
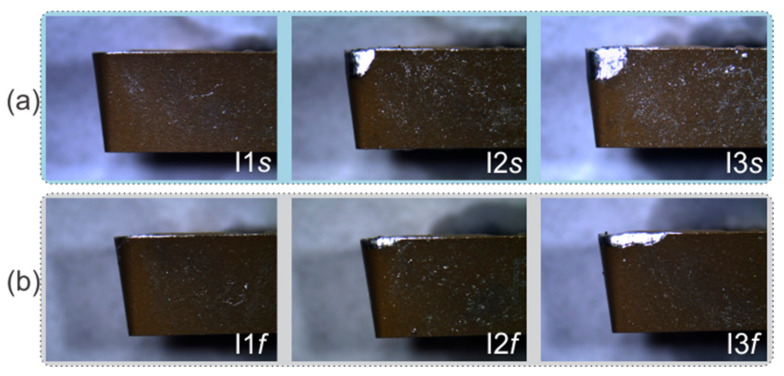
Inserts without wear and with medium and excessive tool wear for experimentation: (**a**) cutting speed, and (**b**) feed rate variation.

For the experiments related to the variation of cutting speed, the three cutting tools used are shown in the images in Figure 8a, taken with a microscope. The tool marked as *I1s* is a new insert with an $A_{f}=0\;{\mathrm{mm}}^{2}$, *I2s* has a medium tool wear with an $A_{f}=1.2645{\;\mathrm{mm}}^{2}$ value, and *I3s* has excessive tool wear with an $A_{f}=2.2814\;{\mathrm{mm}}^{2}$. Table 2 summarizes the five cutting speeds used for the this first set of experiments, while the depth of cut, $a_{p}$, and feed rate, f, were kept constant. The variations were taken from 60 m/min until 100 m/min for each insert, generating a total of 15 tests.

#### 4.2.2. Study Case: Variations in Feed Rate

For the experiments related to the variation of feed rate, the respective three cutting tools used are displayed in the images in Figure 8b, taken with a microscope. Here, the tool named as *I1f* is a new insert with an $A_{f}=0\;{\mathrm{mm}}^{2}$, and the *I2f* and *I3f* inserts have medium and excessive tool wear with $A_{f}=0.5858{\;\mathrm{mm}}^{2}$ and $A_{f}=1.7390\;mm^{2}$ values, respectively. The five feed rates used for the second set of experiments are summarized in Table 3; depth of cut, $a_{p}$, and cutting speed, $v_{c}$, remained constant. The variations were taken from 0.08 mm/rev until 0.24 mm/rev for each insert, generating a total of 15 tests.

### 4.3. Experimental Design for Matrix of Features Computing and Tool-Wear Condition Classification

**Figure 9:**
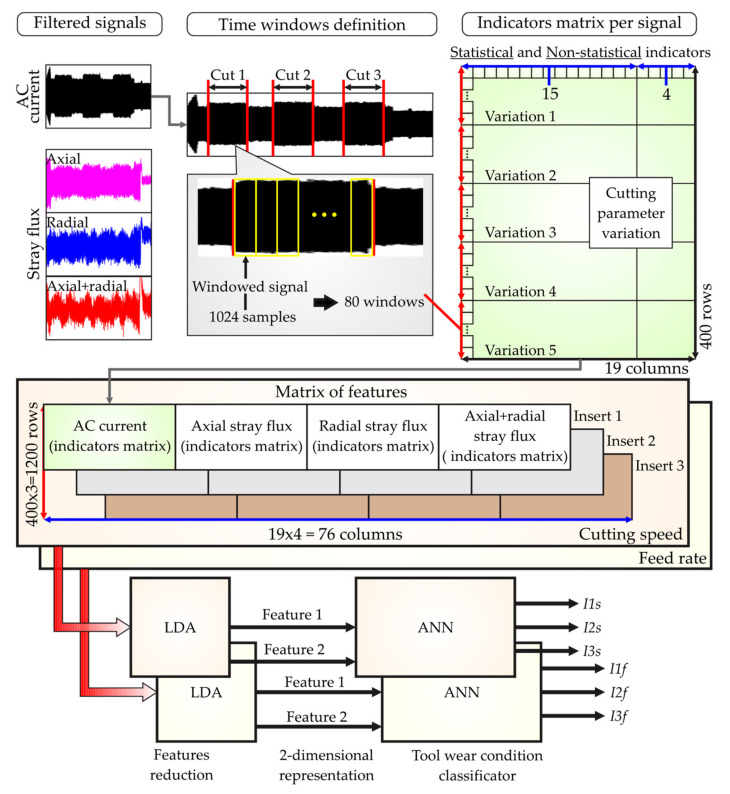
Experimental design for matrix of features computing and tool-wear condition classification.

In summary, for each test, the signals of four sensors were acquired from the spindle motor of the CNC machine (AC current, axial flux, radial flux, and axial + radial flux). For a single signal (for instance, the AC current), the samples of the three cuts were analyzed. For each cut, time-windows of 1024 samples were taken, generating 80 rectangular windowed signals. The advantages of this rectangular function are as follows: it shows better performance than other functions (defined by experimentation), there are a greater number of windows for accurate data analysis, and finally, it is possible to observe small variations in the acquired signals due to the continuous windows taken. Thus, the 19 indicators (statistical and non-statistical) were computed for the 80 windows and for the five variations of a single cutting parameter, creating an indicator matrix with a dimension of 400 × 19. This procedure was repeated for the remaining sensor signals, and their indicator matrices were horizontally concatenated. Once the above was accomplished, the computing of all the indicators for one of the three inserts was complete; the process was then performed twice more for the other two inserts. As a result, a matrix of features of a dimensionof 1200 × 76 was obtained and used as the input to the LDA technique. This technique was configured to reduce the matrix of features into a two-dimensional representation, which meant that only two sets of features (Feature 1 and Feature 2) were obtained, for the three classes stablished, as follows: new insert and inserts with medium and excessive tool wear. Additionally, this reduction helped to reach the maximum separation between the classes defined. The two-dimensional representation was defined in the LDA in order to reduce the computational resources required by yielding only two inputs for the classifier. The classification task of the tool-wear conditions was finally made through a simple ANN topology that consisted of two layers, for which two neurons were used in the input layer, and eight neurons were used for the hidden layer. The activation functions were defined as hyperbolic for the hidden layer and linear for the output. A total of 400 values (80 windows by the five cutting parameter variations) were taken into account, from which 370 and 30 were randomly used without repetition for training and validation of the ANN, respectively. All the procedure were executed twice because variation in cutting speed and feed rate were both considered. Figure 9 presents this experimental design.

## 5. Results

This section presents the results obtained from the experimental tests performed. The following paragraphs are organized by study case, starting with the cutting speed variations and continuing with the feed rate variations. Additionally, in order to demonstrate the effectiveness and robustness of the proposed approach, through the fusion between the AC current sensor signal and the stray flux sensor signals acquired from the spindle motor of the CNC machine, the results are compared with a processing that considers the tool-wear condition using a single variable, which means that AC current and stray flux are analyzed separately. 

### 5.1. Results of Cutting Tool Wear Detection under Variations in Cutting Speed

**Figure 10:**
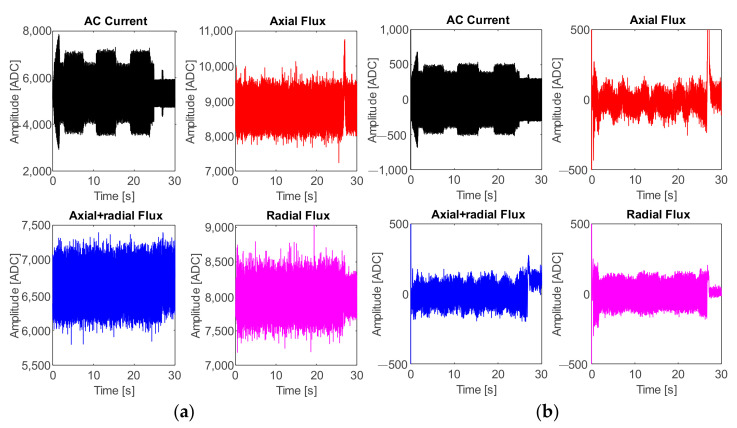
Acquired signals from the four sensors: test taken from variation of cutting speed; (**a**) original signals and (**b**) filtered signals.

The first set of experiments was carried out for the case of the cutting speed variations, and the following plots represent the acquired signals from the four sensors (AC current, axial flux, radial flux, and axial + radial flux) through the DAS module from a single random trial for demonstrative purposes. Thus, for the case of cutting speed, Figure 10a shows the plots of the signals directly obtained from the ADC. Once the low–low pass filter is applied, the obtained signals are those displayed in the plots of Figure 10b, and hereafter these signals are used for the processing stage.

**Figure 11:**
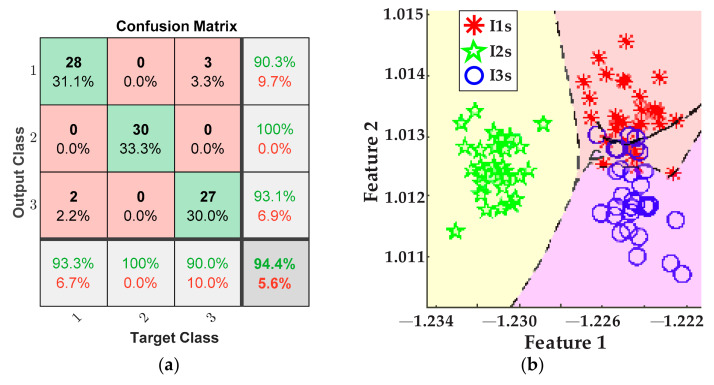
Results from cutting speed variations for the fusion of AC current and stray flux sensors; (**a**) confusion matrix and (**b**) ANN classification.

The plots in Figure 11 depict the results obtained for detecting the tool-wear conditions using the five values of cutting speed from Table 2 for the inserts I1*s*, I2*s*, and I3*s*. These results consider the fusion of features from the AC current demand signal and the stray flux signals coming from the axial, radial, and axial + radial directions of the spindle motor. Figure 11a presents the confusion matrix of the classifier, and Figure 11b shows the classification performed by the ANN. 

**Figure 12:**
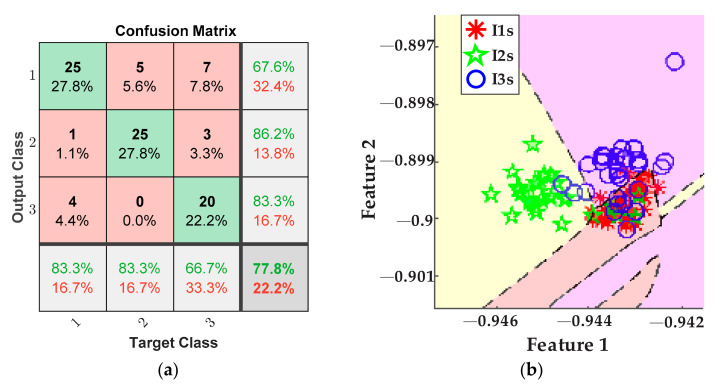
Results from cutting speed variations for the AC current sensor; (**a**) confusion matrix and (**b**) ANN classification.

In Figure 12a, the confusion matrix of the classifier for the tests that consider exclusively the AC current sensor of the spindle motor can be observed, and its respective tool-wear conditions classification through the ANN is plotted in Figure 12b.

**Figure 13:**
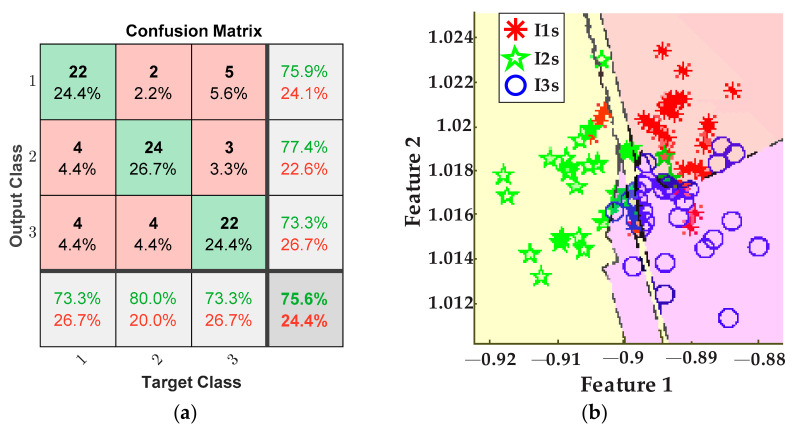
Results from cutting speed variations for the stray flux sensors; (**a**) confusion matrix and (**b**) ANN classification.

In the same way, the last plots of Figure 13 correspond to the tests that only use the stray flux signals coming from the axial, radial, and axial + radial directions of the spindle motor. The confusion matrix can be seen in Figure 13a. In addition, Figure 13b shows the classification of tool-wear conditions obtained by the ANN.

### 5.2. Results of Cutting Tool Wear Detection under Variations in Feed Rate

**Figure 14:**
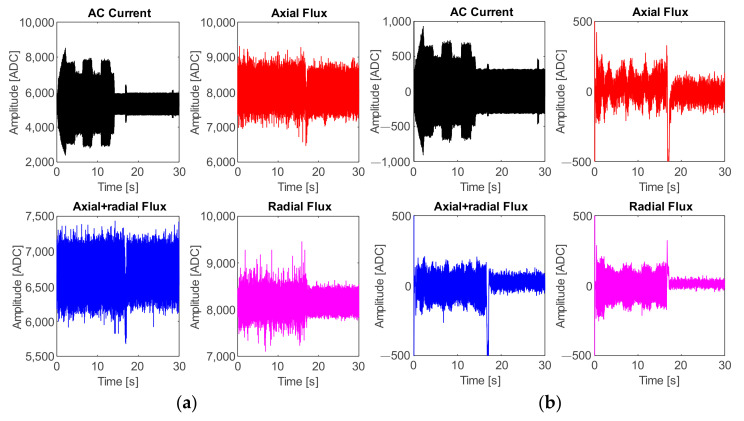
Acquired signals from the four sensors: test taken from variation of feed rate; (**a**) original signals and (**b**) filtered signals.

The second set of experiments was carried out relating to feed rate variations. The following plots represent the acquired signals from the four sensors through the DAS module from a single random trial for demonstrative purposes. Hence, Figure 14a shows the plots of the original signals and Figure 14b displays the filtered signal, which is hereafter used for the processing stage.

**Figure 15:**
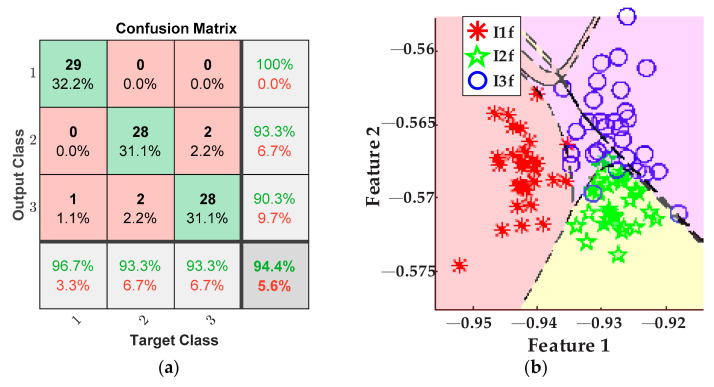
Results from feed rate variations for the fusion of AC current and stray flux sensors; (**a**) confusion matrix and (**b**) ANN classification.

Accordingly, the plots of Figure 15 portray the results obtained for detecting the tool-wear conditions using the five values of feed rate from Table 3 for the inserts *I1f*, *I2f*, and *I3f*. Here, a fusion of features from the AC current demand signal and the stray flux signals coming from the axial, radial, and axial + radial directions of the spindle motor was considered. Thereby, Figure 15a shows the confusion matrix of the classifier, and Figure 15b shows the classification performed by the ANN.

**Figure 16:**
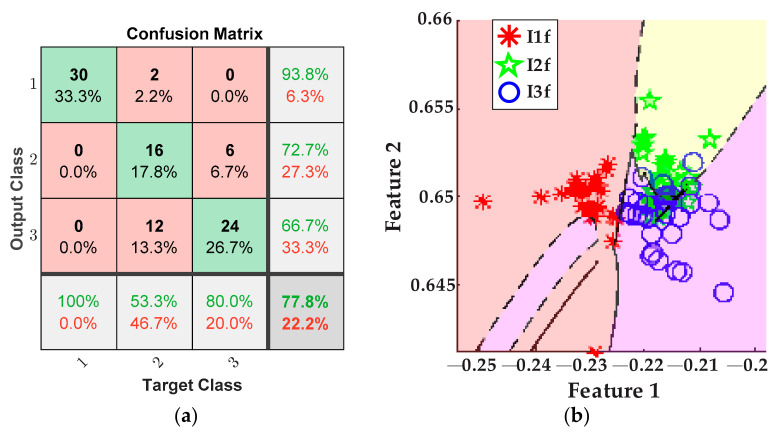
Results from feed rate variations for the AC current sensor; (**a**) confusion matrix and (**b**) ANN classification.

The comparison with the performance when the AC current demand signal of the motor is used is also presented here for the feed rate case. Therefore, Figure 16a displays the confusion matrix of the classifier, and the respective tool-wear conditions classification by the ANN is observed in Figure 16b.

**Figure 17:**
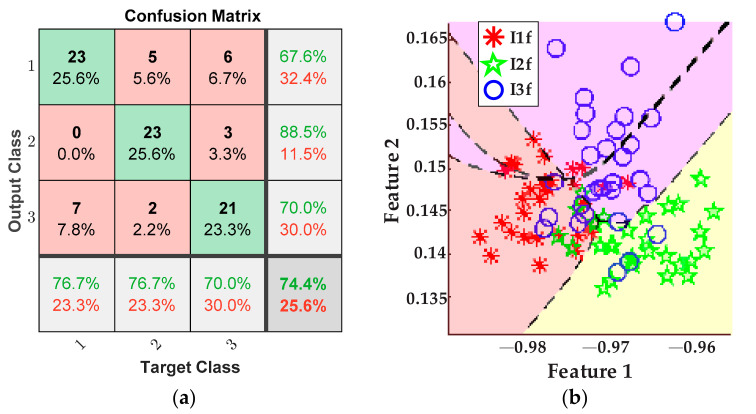
Results from feed rate variations for the stray flux sensors; (**a**) confusion matrix and (**b**) ANN classification.

Again, the final results are for the tool-wear condition detection when only the stray flux signals of the motor are used for processing. They are shown by the plot in Figure 17a, where the confusion matrix can be appreciated. Additionally, Figure 17b shows the classification of tool-wear conditions accomplished by the ANN.

## 6. Discussion

A discussion of the obtained results is presented in this section. Firstly, the importance of the main blocks of the proposed methodology it must be highlighted, starting with the machining process. The variations of the cutting parameters are important because they provide the methodology for a matrix of experiments with several conditions that could be related to the tool-wear condition. For example, the variation in the cutting speed and the feed rate are related to the amount of current and stray flux demanded and generated, respectively, on the spindle motor. The physical variables must then be captured; here, the DAS module plays a key role, because data must be sufficient and has to be acquired at a convenient sampling frequency that allows the definition of enough windowed signals to compute the necessary mathematical indicators. For instance, a matrix of features must contain a high number of values in order to extract those features that yield an effective tool-wear condition classification. 

From the obtained results, for the case of cutting speed variation, it can be observed that tool-wear condition is successfully detected and categorized by fusing the AC current signal and the stray flux signals detected by the sensors mounted in the spindle motor, and by means of the statistical and non-statistical features, extracted and reduced, that better describe the behavior of the sampled data. Specifically, by analyzing the plots of the study case where the cutting speed is changed, Figure 11a demonstrates an excellent performance in differentiating between the three tool-wear conditions, *I1s*, *I2s*, and *I3s*, because a general 94.4% is finally reached. Indeed, only two errors in the diagnostics occur when two data of condition *I1s* were considered as condition *I3s*, and three data of condition *I3s* were considered as condition *I1s* from the 30 data of validation. In parallel, condition *I2s* was successfully characterized without errors. The tool-wear classification of the ANN is showed in Figure 11b; here, only a very slight overlapping between condition *I1s* and condition *I3s* is observed. Nevertheless, in comparison with the results of tool-wear condition classification made by processing only the AC current demand signal of the spindle motor, Figure 12a, it is noticed that the overall performance for these tests is only 77.8%, which is noticeably lower than the results obtained from the fusion of sensors. This means that, even considering and computing all the same 19 indicators for the acquired current signals, the information contained in the matrix of features is not enough for making a good discrimination between the different tool wear presented in the inserts. Additionally, it should not be forgotten that by using only the AC current signal, the dimension of the matrix of features is reduced to 1200 × 19, leaving a lower number of values to evaluate. In consequence, a good deal of valuable information is not considered, and then, in Figure 12b, a greater overlapping between conditions *I1s* and *I3s* is observed; a moderate overlapping even occurs with condition *I2s*. Similarly, a comparison can be made with respect to the results of tool-wear condition classification made by using only the stray flux signals, Figure 13a, where 75.6% is reached. Here, the flux signals are acquired from the axial, radial, and axial + radial directions of the spindle motor, yielding a dimension of 1200 × 57 for the matrix of features. Evidently, this information is not sufficient to reach a better performance, Figure 13b; additionally, the matrix has the same quality and influence as the AC current signal matrix, because a similar percentage is obtained. This observation can be justified because the three plots of stray flux do not show a good definition in the machining cuts of the trial, in contrast with the AC current signal, which is visibly better, Figure 10b.

Other points to discuss are the results for tool-wear condition for the case feed rate variation. Equally, the tool-wear conditions of inserts *I1f*, *I2f*, and *I3f* are successfully detected and categorized by fusing the AC current signal and the stray flux signals acquired from the spindle motor. In this case, Figure 15a demonstrates excellent performance in differentiating between the three tool-wear conditions because an overall 94.4% is reached. In this case, only three errors in the diagnostics occur when one datum of condition *I1f* was considered as condition *I3f*, two data of condition *I2f* were considered as condition *I3f*, and two data of condition *I3f* were considered as condition *I2f* from the 30 data of validation. Furthermore, the tool-wear classification of the ANN is showed in Figure 15b; here, only a very slight overlapping between condition *I2f* and condition *I3f* is observed. In the same way, Figure 16a allows a comparison with the results of the classification made by processing only the AC current signal of the motor; it is observed that the performance for these tests barely reaches 77.8%, which is notably under the percentage reached for the fusion of sensors. Additionally, by taking into account only the AC current, the dimension of the matrix of features is reduced to 1200 × 19. As result, much valuable information is not considered and then a strong overlapping between conditions *I2f* and *I3f* is observed, Figure 16b. Finally, Figure 17a displays a lower performance from the results of the tool-wear condition classification, made through the stray flux signals of the spindle motor only, where 74.4% is reached in comparison with the fusion of sensors. Here, the dimension of the matrix of features is only 1200 × 57. The performance percentage for the analysis of stray flux is similar to the performance percentage for the analysis of the AC current; therefore, the same justification related to the quality of the signals is taken. This means that the three signals of stray flux have the same impact as the AC current signal, Figure 14b.

## 7. Conclusions

This work develops a novel methodology that uses the signal of AC current and the signals of stray flux coming from the axial, radial, and axial + radial directions from the spindle motor of a CNC lathe for detecting and classifying tool-wear condition under cutting parameter variation (cutting speed and feed rate). The proposed methodology here described looks to take advantage of each physical variable (AC current and stray fluxes) to achieve an excellent performance in the detection and classification of tool-wear conditions. To this end, two sets of three inserts were analyzed, each set with three tool-wear conditions, the first set under cutting speed variation and the second set under feed rate variation.

For the experimental trials, workpieces of aluminum 6061 were used in a Fanuc Oi Mate CNC lathe, and the displayed results demonstrate excellent tool-wear condition detection and classification, reaching performances up to 94.4% for both study cases: cutting speed and feed rate. It is worth mentioning that methodologies that apply the fusion of sensors allow an increase in the overall performance in comparison with methodologies that use single sensor signals. For this work, in the cutting speed variation, the fusion of sensors in the proposed methodology has a performance of 16.6% above the performance using AC current signals, and 18.8% above the performance using stray flux signals. Meanwhile, in the feed rate variation, the fusion of sensors has a performance of 16.6% above the performance using AC current signals, and 20% above the performance using stray flux signals. It is also concluded that feature extraction from signals of physical variables using statistical and non-statistical indicators is very helpful. To include them in a matrix of features to finally make a dimension reduction in order to apply intelligent classification technique is a good tool. However, the kind of features that are expected to be analyzed from the matrix must be considered. For instance, statistical indicators help to provide information regarding the means, distributions, and tendences of data. Meanwhile, non-statistical indicators such as HFD, KFD, $\gamma_{DWT}$, and $S_{WT}$ help to provide other information, such as the amount of self-similarity or repeated patterns, energy changes in a given signal, and the entropy of a signal, respectively. With this in mind, the quality of the matrix of features is reinforced in this work using a wide range of information. The main drawbacks in the development of this work were not being able to experiment with other types of CNC machines and under other external parameter variations, such as the chip load and speed. However, access to laboratories for experimentation was restricted by the SARS- CoV-2 pandemic around the world, which is the main reason this investigation has being realized only gradually, with limited parameters. In future work, we expect to address other conditions in the machining process, along with cutting parameters. Finally, future works will involve looking at detecting and classifying other anomalies in the machining process by considering other non-invasive physical variables such as sound. Additionally, the matrix of features could be optimized by hybridizing with other indicators, increasing the number of dimensions, and applying metaheuristic techniques. Additionally, it would be interesting to prove if the proposed methodology is insensible for tool-wear condition monitoring when it is applied in CNC machines with motors that have different efficiencies.

## Figures and Tables

**Table 1:** Proposed set of statistical time-domain features for the characterization of the dynamic behavior of a signal.

Statistical Time-Domain Feature	Mathematical Equation	
Mean	$T_{1}=\frac{1}{N}\cdot \sum_{i=1}^{N}\left|x_{i}\right|$	(4)
Maximum value	$T_{2}=max\left(x\right)$	(5)
Root mean square	$T_{3}=\sqrt{\frac{1}{N}\cdot \sum_{i=1}^{N}{\left(x_{i}\right)}^{2}}$	(6)
Square root mean	$T_{4}={\left(\frac{1}{N}\cdot \sum_{i=1}^{N}\sqrt{\left|x_{i}\right|}\right)}^{2}$	(7)
Standard deviation	$T_{5}=\sqrt{\frac{1}{N}\cdot \sum_{i=1}^{N}{\left(x_{i}-T_{1}\right)}^{2}}$	(8)
Variance	$T_{6}=\frac{1}{N}\cdot \sum_{i=1}^{n}{\left(x_{i}-T_{1}\right)}^{2}$	(9)
RMS Shape factor	$T_{7}=\frac{T_{3}}{\frac{1}{N}\cdot \sum_{i=1}^{N}\left|x_{i}\right|}$	(10)
SRM Shape factor	$T_{8}=\frac{T_{4}}{\frac{1}{N}\cdot \sum_{i=1}^{N}\left|x_{i}\right|}$	(11)
Crest factor	$T_{9}=\frac{T_{2}}{T_{3}}$	(12)
Latitude factor	$T_{10}=\frac{T_{2}}{T_{4}}$	(13)
Impulse factor	$T_{11}=\frac{T_{2}}{\frac{1}{N}\cdot \sum_{i=1}^{N}\left|x_{i}\right|}$	(14)
Skewness	$T_{12}=\frac{\sum \left[{\left(x_{i}-T_{1}\right)}^{3}\right]}{T_{5}^{3}}$	(15)
Kurtosis	$T_{13}=\frac{\sum \left[{\left(x_{i}-T_{1}\right)}^{4}\right]}{T_{5}^{4}}$	(16)
Fifth moment	$T_{14}=\frac{\sum \left[{\left(x_{i}-T_{1}\right)}^{5}\right]}{T_{5}^{5}}$	(17)
Sixth moment	$T_{15}=\frac{\sum \left[{\left(x_{i}-T_{1}\right)}^{6}\right]}{T_{5}^{6}}$	(18)

**Table 2:** Cutting parameters for cutting speed variations.

**No**	**Insert Tool**	$a_{p}\left(mm\right)$	$f\left(mm/rev\right)$	$v_{c}\left(m/rev\;\right)$	$N\left(rpm\right)$	$V_{f}\left(mm/min\right)$
1	*I1s*, *I2s*, *I3s*	1.25	0.16	60	779.53	124.72
2		1.25	0.16	70	909.45	145.51
3		1.25	0.16	80	1039.37	166.30
4		1.25	0.16	90	1169.29	187.08
5		1.25	0.16	100	1299.22	207.87

**Table 3:** Cutting parameters for feed rate variations.

**No**	**Insert Tool**	$a_{p}\left(mm\right)$	$f\left(mm/rev\right)$	$v_{c}\left(m/rev\;\right)$	$N\left(rpm\right)$	$V_{f}\left(mm/min\right)$
1	*I1f*, *I2f*, *I3f*	1.25	0.08	100	1299.22	103.938
2		1.25	0.12	100	1299.22	155.907
3		1.25	0.16	100	1299.22	207.875
4		1.25	0.20	100	1299.22	259.844
5		1.25	0.24	100	1299.22	311.813

## Data Availability

No new data were created or analyzed in this study. Data sharing is not applicable to this article.
